# Potential gains by effective early detection of diseases: proposal to approach informing public health policy in the Czech Republic

**DOI:** 10.1093/eurpub/ckac129.201

**Published:** 2022-10-25

**Authors:** O Majek, O Ngo, K Hejduk

**Affiliations:** 1 Institute of Health Information and Statistic, Prague, Czechia; 2 Institute of Biostatistics and Analyses, Prague, Czechia

## Abstract

**Background:**

Significant amount of disease burden could be averted by early detection and treatment of diseases. In the Czech Republic, National Screening Centre (NSC) of the Institute of Health Information and Statistics is responsible for informing public health policy in the field of early disease detection. The objective of the proposed early detection public health foresight study (PHFS) is to gather evidence, use available computational tools, utilise knowledge and opinions of stakeholders, and summarize it in a systematic manner to inform public health policies. The presentation outlines the approach undertaken within the proposed early detection PHFS.

**Methods:**

The key source of data for monitoring of population health status and healthcare system in the Czech Republic is the National Health Information System (NHIS). The study will also utilise external sources of data, namely demographic projections and data on global burden of disease, as well as qualitative data from stakeholders. The study will also utilise analytical tools and outputs developed by NSC (situational analyses, decision modelling, etc.). The conceptual model of the study will cover important underlying aspects like public policies, driving forces, population health and healthcare system, and health impact variables.

**Results:**

The proposal for early detection PHFS has been developed within the PHFS capacity building course, utilising the experience and insights of tutors and fellow participants. The development of study methodology was accompanied by gathering of evidence and consultations with relevant stakeholders.

**Conclusions:**

PHFS is a very useful approach to assess possible future developments of public health system, achieve participation of stakeholders, and to inform public health strategies. Following finalisation of the early detection PHFS protocol, governing board of the NSC will decide on the degree of implementation of the study.

